# Supplementary material for: Integrated analyses of murine breast cancer models reveal critical parallels with human disease
Source: Nat Commun. 2019 Jul 22;10:3261. doi: 10.1038/s41467-019-11236-3 (PMC6646342; doi:10.1038/s41467-019-11236-3)
Supplement: Supplementary file 1 — Supplementary Information [file 41467_2019_11236_MOESM1_ESM.pdf]

RENNHACK - Figure S1

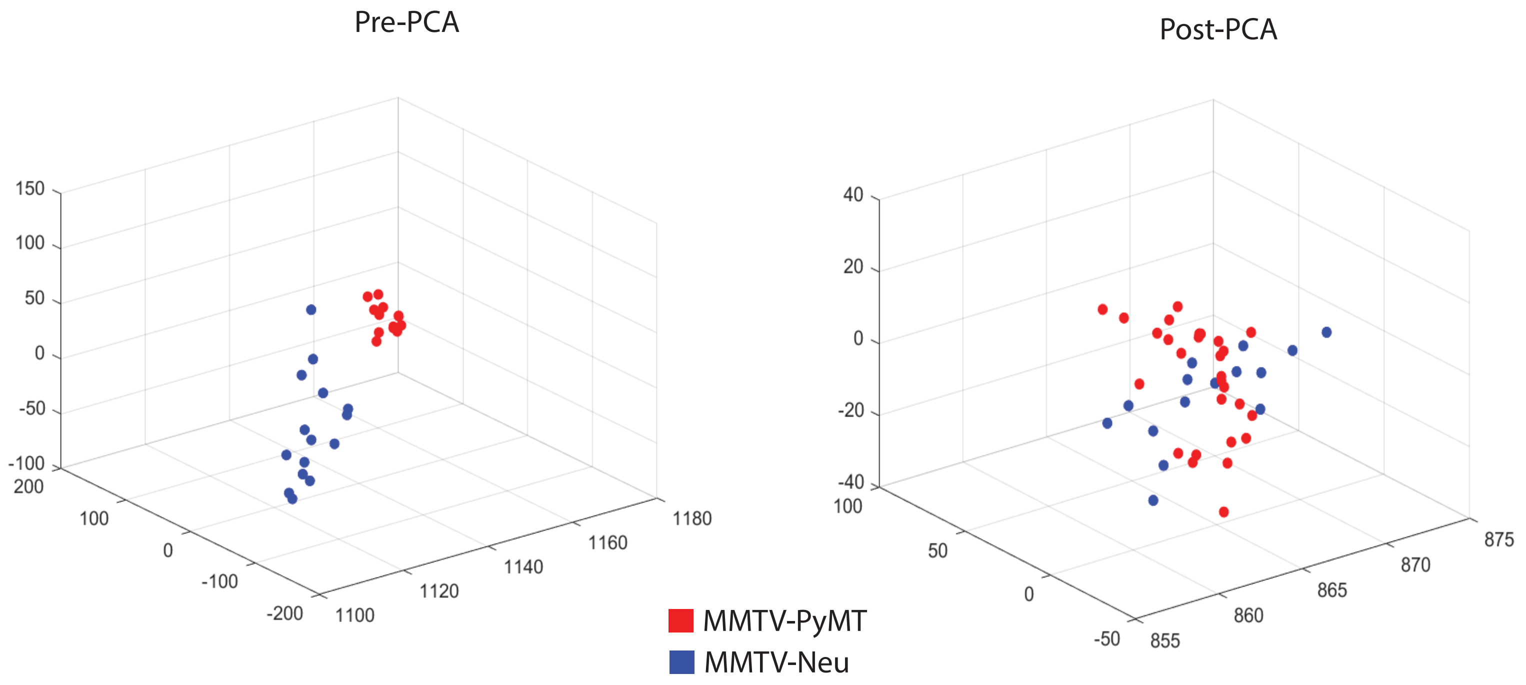

Supplementary Figure 1: Batch effect correction

PCA plots of the batch effect correction before (A) and after (B) the BFRM batch effect corrections (Red – MMTV-PyMT, Blue – MMTV-Neu)

RENNHACK - Figure S2

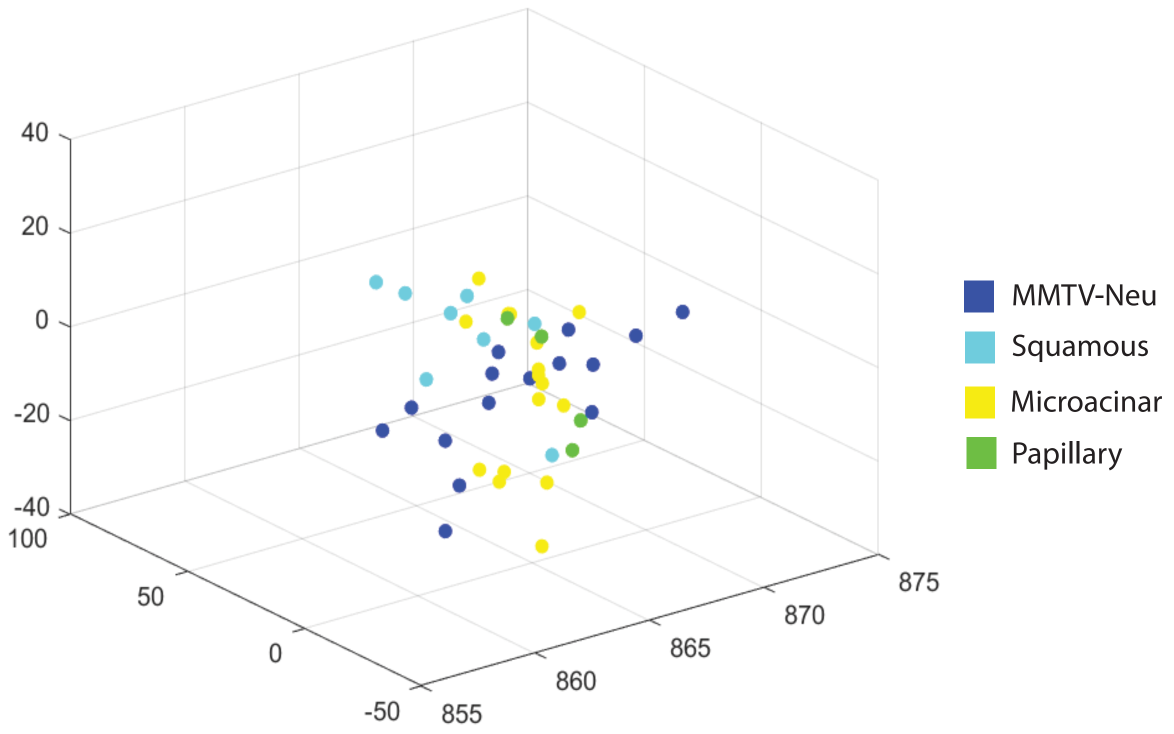

Supplementary Figure 2: PCA analysis of models by histological subtypes

PCA analysis shows distinct signaling pathways between the MMTV-Neu (blue) and each of the histological subtypes present in the PyMT model (cyan – squamous, yellow – microacinar, green – papillary)

RENNHACK - Figure S3

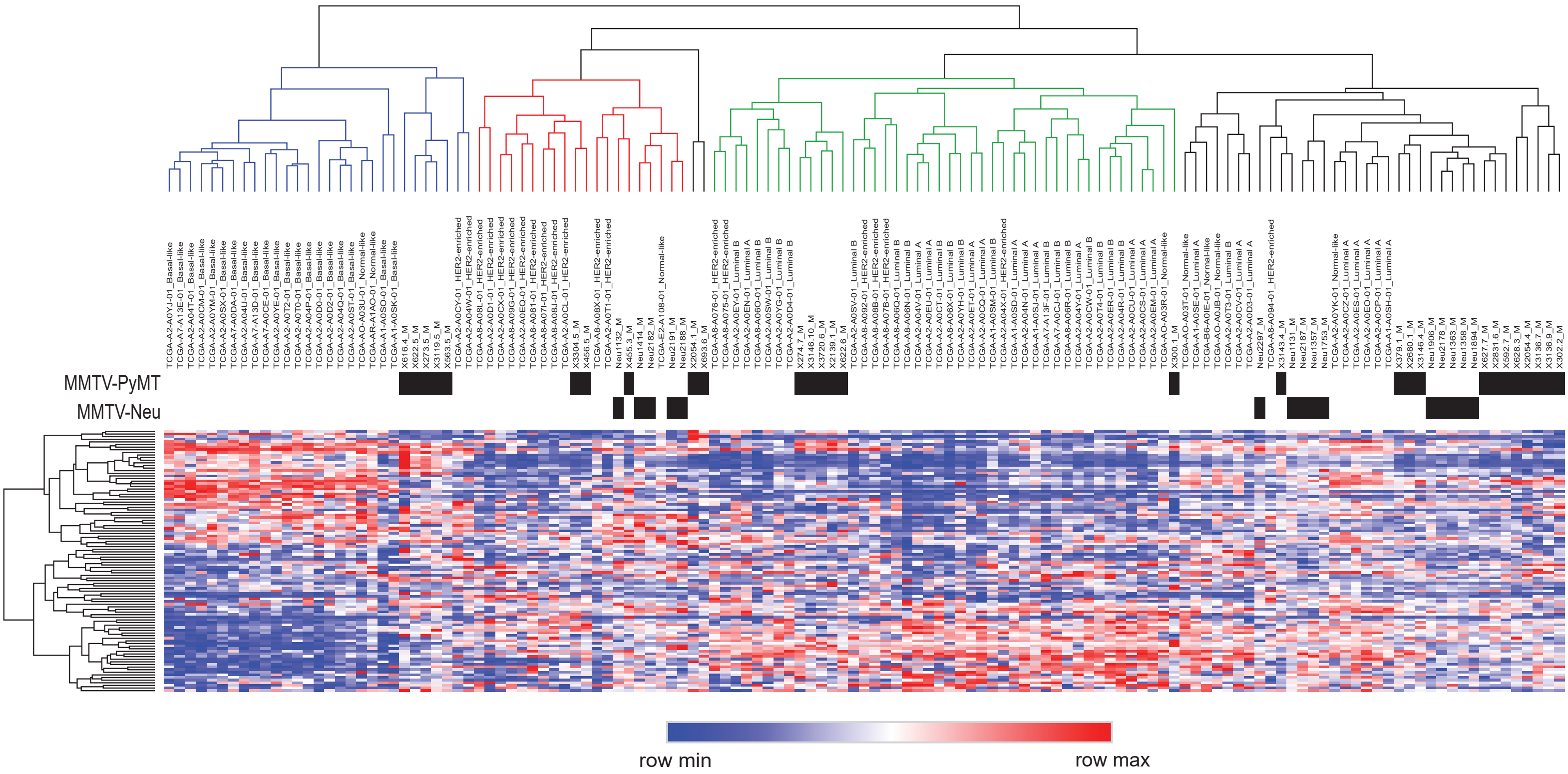

Supplementary Figure 3: Heatmap representing co-clustering of breast cancer and each mouse model

Unsupervised hierarchical clustering shows co-clustering between human and mouse tumors. Individual tumors are represented by individual columns and genes by row, expression is signified by color (red – high, blue – low). It was seen that many of the tumors clustered into a mouse specific expression profile (black cluster). Despite this there was some overlap between the MMTV-PyMT and basal like tumors (blue cluster), both models and the HER2+ tumors (red cluster), and MMTV-PyMT and luminal tumors (green cluster).

RENNHACK - FIGURE S4

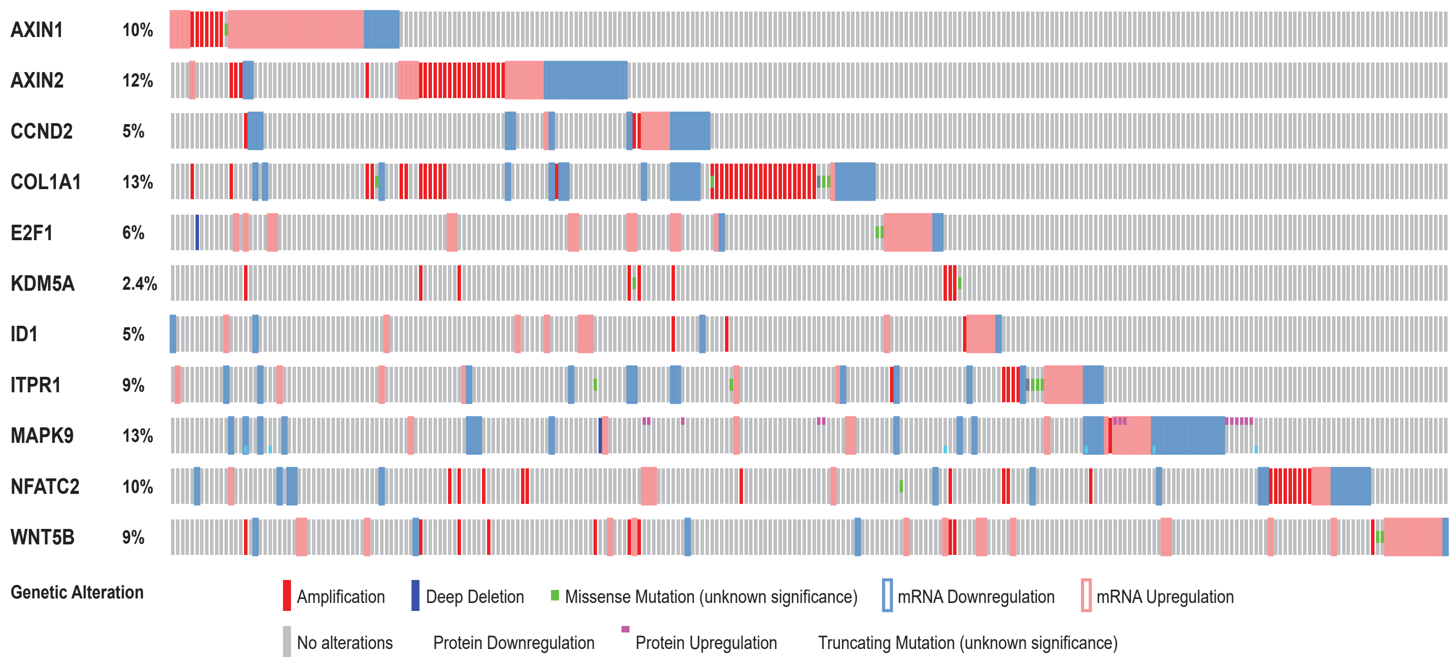

Supplementary Figure 4: Copy number alterations TCGA Breast Cancer Oncoprint

Oncoprint of the human TCGA Breast cancer cohort (Nature 2012) displaying the alteration of genes altered at a high rate in mouse models with regards to copy number

RENNHACK - FIGURE S5

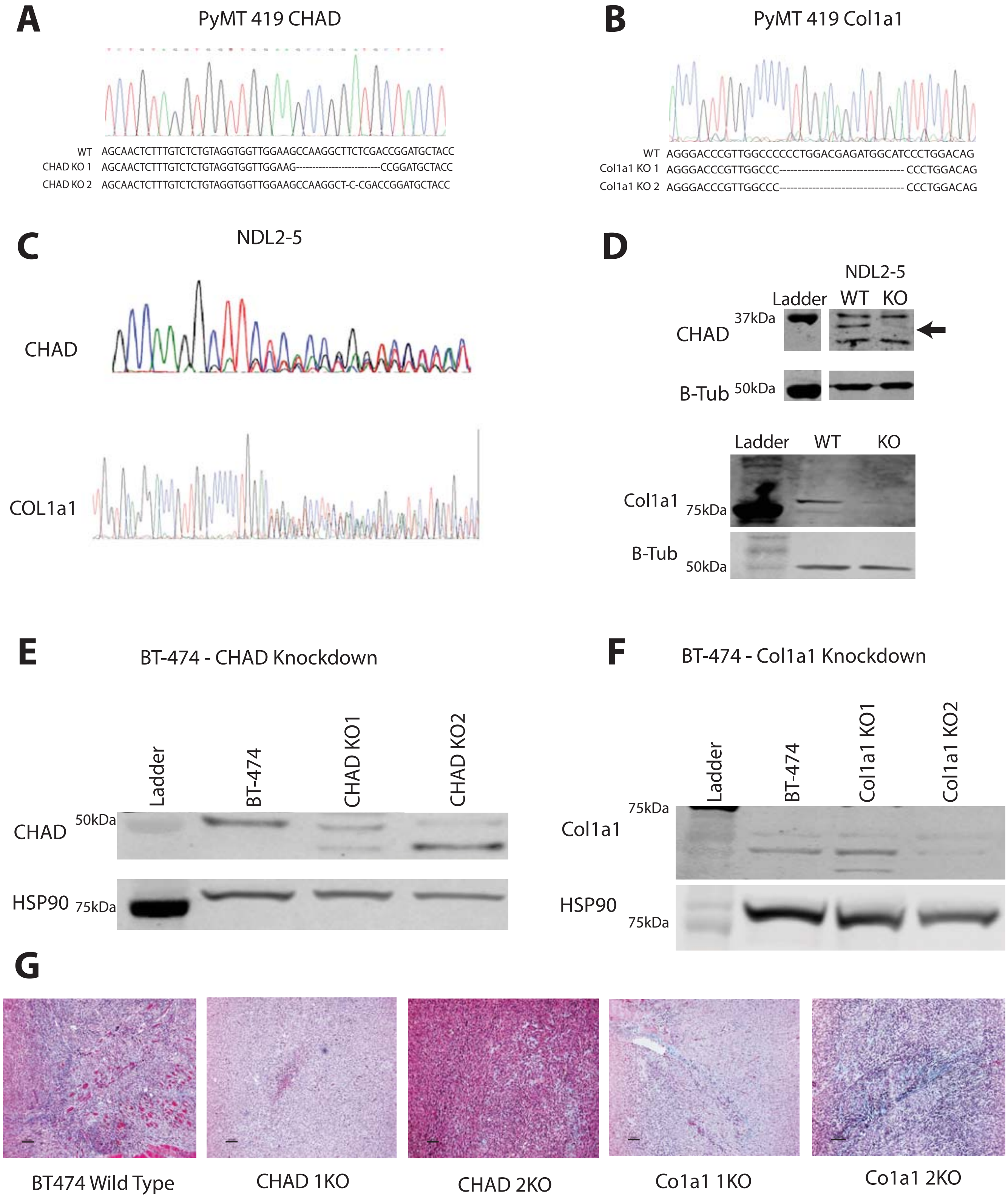

Supplementary Figure 5 - Confirmation of Col1a1 and CHAD knockout in PyMT 419, NDL2-5, and BT-474 cell lines
Sanger sequencing of CHAD (A) and Col1a1 (B) KO clones revealed the production of indels within the coding sequence of each protein within the PyMT 419 line. This is also the case where multiple different indels were shown in the Col1a1/CHAD amplified cell line NDL2-5 (C). The confirmation of knockdown was completed through western blot for CHAD (top) and Col1a1 (Bottom). The CRISPRi system with guides against early exons of CHAD and Col1a1 was used to generate knockdowns of the respective genes in the human HER2 positive, COL1A1/CHAD amplified line BT474. The efficiency of knockdown in the pooled population was assessed through western blot for CHAD (E) and COL1A1 (F). It was also seen that the production of collagen fibers was impaired in the CRISPRi knockdown population through the use of Masson's trichrome. Scale bar of 20 um displayed on image for reference (G).

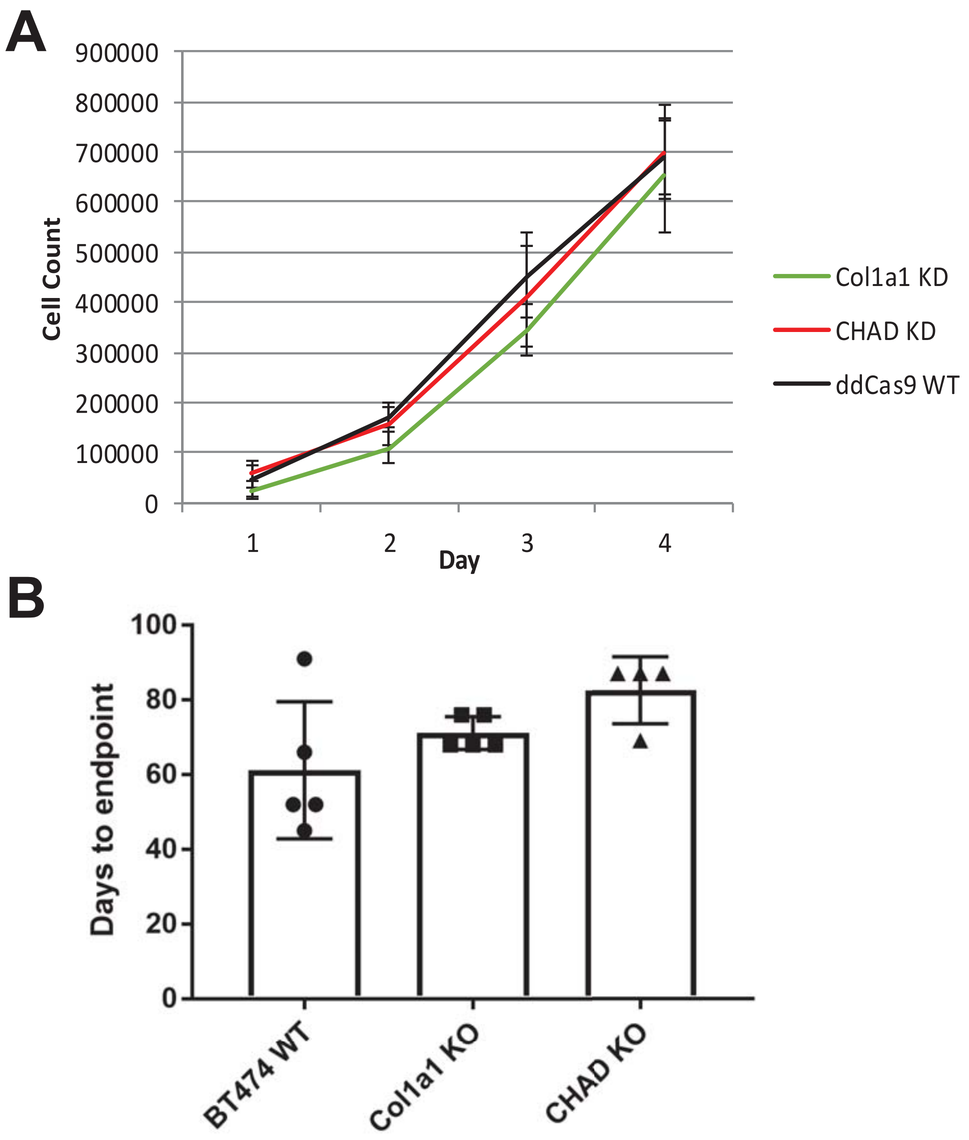

Supplementary Figure 6 – Growth rate impact of loss of Col1a1 and CHAD

There were no detected defects in growth with the loss of Col1a1 of CHAD in either the *in vitro* (A) or *in vivo* model (B) (P>.05, students two-tailed, unpaired t-test).

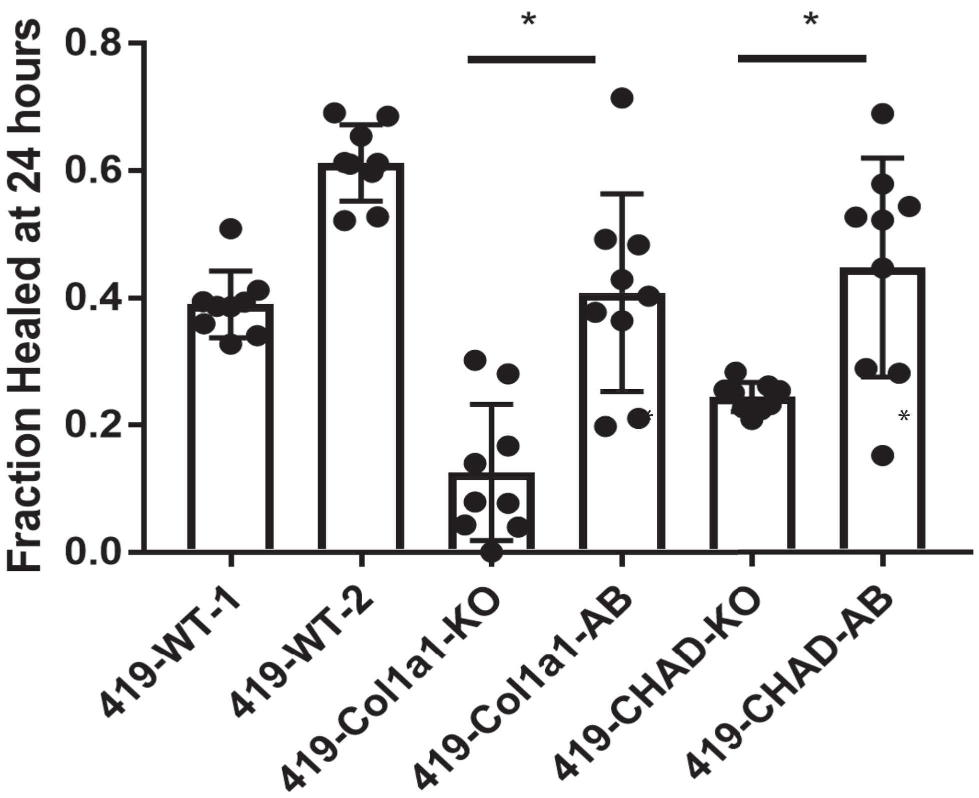

Supplementary Figure 7 - Addback of Col1a1 and CHAD in PyMT 419 cell lines

The addback of wildype *Col1a1* and *Chad* into the CRISPR generated knockout lines showed partial recovery of movement in a scratch assay (*=P<.05, students two-tailed, unpaired t-test).

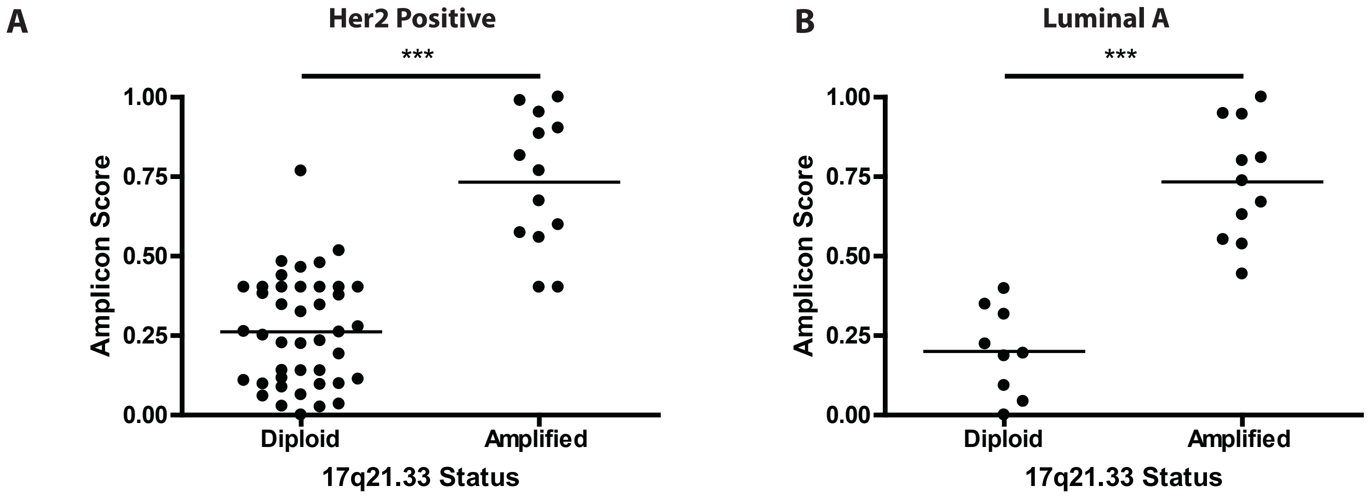

Supplementary Figure 8 - Validation of COL1A1/CHAD amplicon gene expression signature
A score between 0 (diploid) and 1 (amplified) was generated for the predicted presence of the COL1A1/CHAD amplification event based upon a weighted gene expression data. This signature showed a robust prediction of the amplification event in both the training HER2 positive dataset (A) and the Luminal A validation cohort (B)

RENNHACK- Figure S9

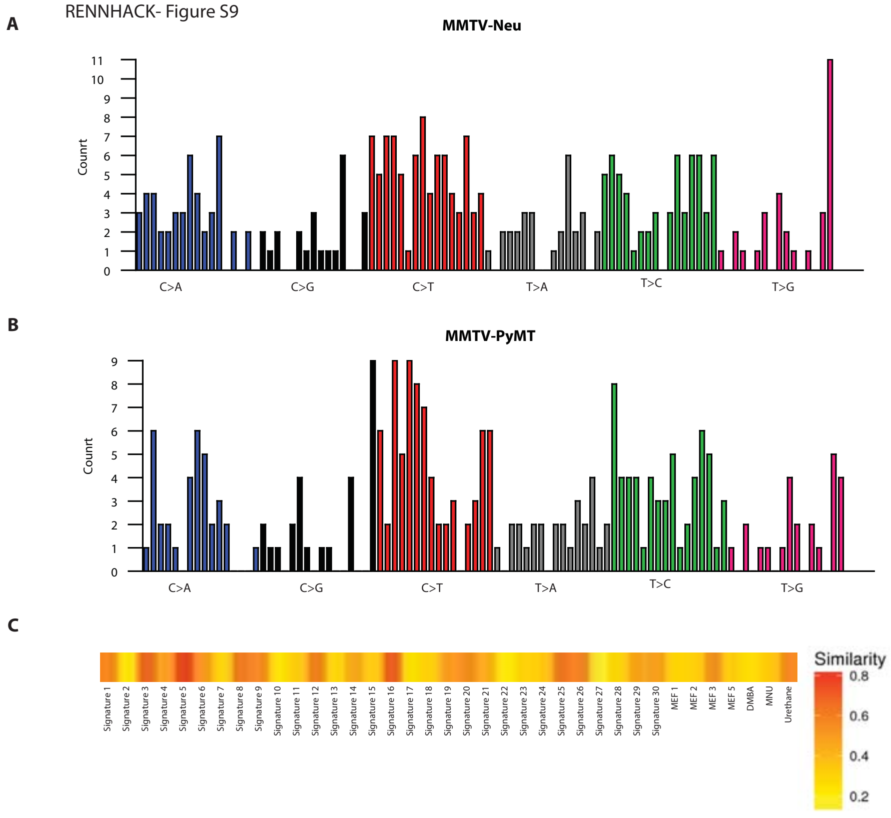

Supplementary Figure 9 - Mutational Signatures of MMTV-Neu and MMTV-PyMT Models

The trinucleotide context of MMTV-Neu (A) and MMTV-PyMT (B) samples are similar. They show the presence of every mutation possibility with the overrepresentation of the C>T and T>C transitions. These trinucleotide signatures were compared with human mutational signatures through the use of a Baysian model high similarity (Red) and low similarity (yellow) were identified through the use of a heat map (C). Signature 5 represented the highest similarity score.

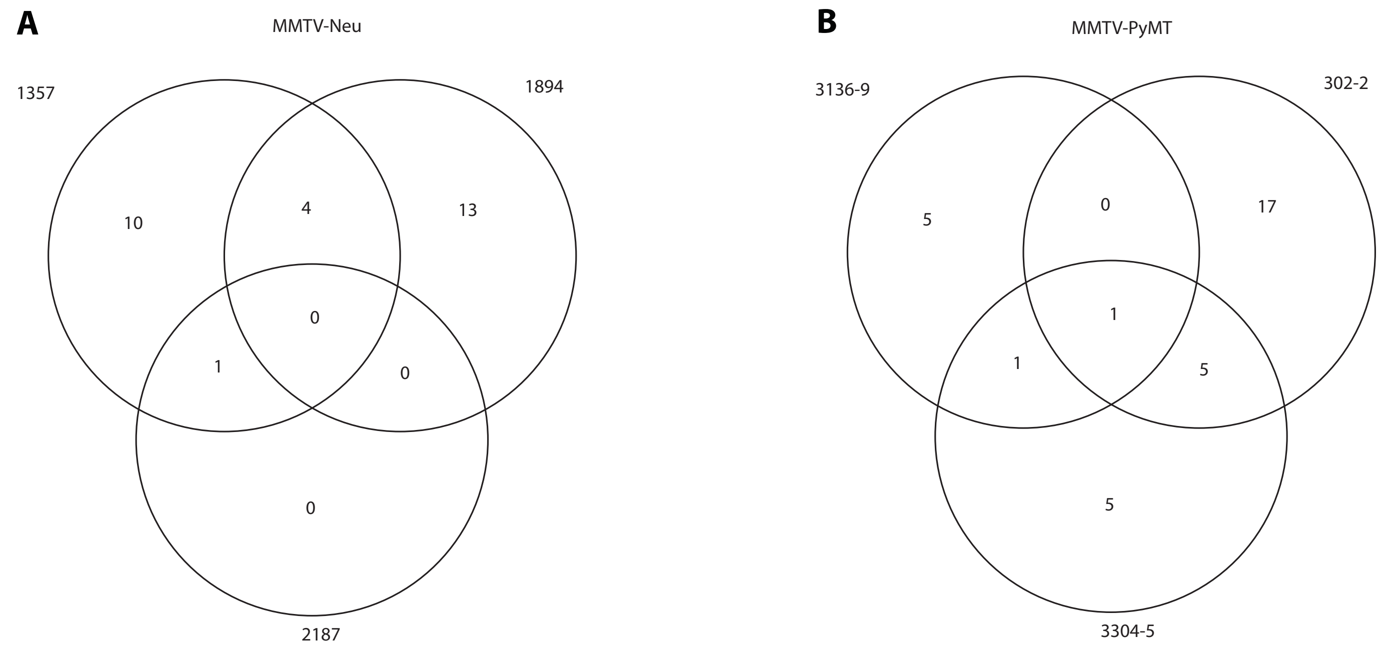

Supplementary Figure 10 - Heterogeneity of SNVs in mouse models of breast cancer

The MMTV-Neu (A) and MMTV-PyMT (B) models have considerable diversity in regards to SNVs. Samples were analyzed for overlap in SNV calls through the use of a Venn Diagram
