# Supplementary material for: Integrated analyses of murine breast cancer models reveal critical parallels with human disease
Source: Nat Commun. 2019 Jul 22;10:3261. doi: 10.1038/s41467-019-11236-3 (PMC6646342; doi:10.1038/s41467-019-11236-3)
Supplement: Supplementary file 3 — Description of Additional Supplementary Files [file 41467_2019_11236_MOESM3_ESM.pdf]

Description of Additional Supplementary Files

File Name: Supplementary Data 1
Description: A table listing the characteristics of tumors used in this study including, model, latency, histological subtype, and number of lung metastases

File Name: Supplementary Data 2
Description: The table contains the PCR and qPCR validation of the bioinformatic calls of SNVs and amplification events. Alterations labeled as WT were not identified as altered in copy number or SNV by the variant callers. These were tested to control for false positives

File Name: Supplementary Data 3
Description: A table listing called SNVs within the sequenced MMTV-PyMT and MMTV-Neu samples. This table also contains the predicted impact of the SNVs on the protein content and function

File Name: Supplementary Data 4
Description: A table listing called copy number alterations within the sequenced MMTV-PyMT and MMTVNeu samples and identification of commonly amplified or deleted genes

File Name: Supplementary Data 5
Description: A table listing called translocations within the sequenced MMTV-PyMT and MMTV-Neu samples

File Name: Supplementary Data 6
Description: A list of gene names and weights that correlate with Col1a1/CHAD amplification generated through WGCNA analysis which are used to create the predictive Col1a1/CHAD amplification gene expression signature

File Name: Supplementary Data 7
Description: Table containing all oligo sequences used for the PCR reaction and sgRNAs in this manuscript
