# Supplementary material for: Integrated analyses of murine breast cancer models reveal critical parallels with human disease
Source: Nat Commun. 2019 Jul 22;10:3261. doi: 10.1038/s41467-019-11236-3 (PMC6646342; doi:10.1038/s41467-019-11236-3)
Supplement: Supplementary file 11 — Reporting Summary [file 41467_2019_11236_MOESM11_ESM.pdf]

nature research

Corresponding author(s): Eran Andrechek

Last updated by author(s): May 12, 2019

Reporting Summary

Nature Research wishes to improve the reproducibility of the work that we publish. This form provides structure for consistency and transparency in reporting. For further information on Nature Research policies, see Authors & Referees and the Editorial Policy Checklist.

Statistics

For all statistical analyses, confirm that the following items are present in the figure legend, table legend, main text, or Methods section.

n/a	Confirmed	
☐	☒	The exact sample size (*n*) for each experimental group/condition, given as a discrete number and unit of measurement
☐	☒	A statement on whether measurements were taken from distinct samples or whether the same sample was measured repeatedly
☐	☒	The statistical test(s) used AND whether they are one- or two-sided<br>*Only common tests should be described solely by name; describe more complex techniques in the Methods section.*
☒	☐	A description of all covariates tested
☒	☐	A description of any assumptions or corrections, such as tests of normality and adjustment for multiple comparisons
☐	☒	A full description of the statistical parameters including central tendency (e.g. means) or other basic estimates (e.g. regression coefficient) AND variation (e.g. standard deviation) or associated estimates of uncertainty (e.g. confidence intervals)
☐	☒	For null hypothesis testing, the test statistic (e.g. *F*, *t*, *r*) with confidence intervals, effect sizes, degrees of freedom and *P* value noted<br>*Give P values as exact values whenever suitable.*
☒	☐	For Bayesian analysis, information on the choice of priors and Markov chain Monte Carlo settings
☒	☐	For hierarchical and complex designs, identification of the appropriate level for tests and full reporting of outcomes
☐	☒	Estimates of effect sizes (e.g. Cohen's *d*, Pearson's *r*), indicating how they were calculated

Our web collection on statistics for biologists contains articles on many of the points above.

Software and code

Policy information about availability of computer code

Data collection

No code was used for collection of data

Data analysis

Sable software was used for the analysis of data and described below.

Transcriptomic profiling
Transcriptome data for this study was previously published. Data was downloaded from GSE42533 (MMTV-Neu) and GSE104397 (MMTV-PyMT) as .cel files. Affymetrix expression console was used to normalize each individual dataset using RMA normalization. To remove batch effects between datasets BRFM normalization33 was performed with standard parameters and visualized with PCA.
Clustering
Unsupervised hierarchical clustering was performed using Cluster 3.0 and the Broad institute’s Morpheus interface. Heatmaps were created using the MATLAB imagesc function.
Variant calling
Generated .fastq files were assessed for quality control using FASTQC analysis (http://www.bioinformatics.babraham.ac.uk/projects/fastqc). Reads were trimmed for quality using Trimmomatic. After trimming, data was reassessed for quality using FASTQC. Then reads were aligned to the mm10 mouse reference genome using BWA-mem. After alignment, base recalibration and pcr induced biases were removed using PICARD tools (http://broadinstitute.github.io/picard). For variant calling we utilized four software packages, GATK, Mutect2, Strelka, and SomaticSniper. To be a legitimate variant we filtered to only those variants called by 3 of the 4 packages. To control for differences in the FVB strain and the mm10 reference genome we used previously published normal FVB tissue (ERR046395). To call copy number and structural variants we used Delly. For copy number we used default quality control settings and only analyzed those copy number events which had precise boundaries and were larger than 100KB. For translocations we used default quality control setting and precise breakpoints.

Circos visualization
Representative MMTV-Neu and MMTV-PyMT samples were chosen to be displayed as CIRCOS plots. CIRCOS plots were generated using CIRCOS v 0.69 and SNVs, CNVs, and translocations were mapped according to their location on the mm10 genome.

Mutation signatures
Due to the low mutational burden of MMTV-Neu and MMTV-PyMT tumors, mutations were combined into a signal analysis for each model. These samples were processed with MutSpec-NMF for trinucleotide context and comparison to the known human mutation signatures.

For manuscripts utilizing custom algorithms or software that are central to the research but not yet described in published literature, software must be made available to editors/reviewers. We strongly encourage code deposition in a community repository (e.g. GitHub). See the Nature Research guidelines for submitting code & software for further information.

Data

Policy information about availability of data

All manuscripts must include a data availability statement. This statement should provide the following information, where applicable:

- Accession codes, unique identifiers, or web links for publicly available datasets
- A list of figures that have associated raw data
- A description of any restrictions on data availability

The datasets generated during and/or analyzed during the current study are available in the GEO and SRA repositories. The fastq data, for the MMTV-Neu and MMTV-PyMT models is publicly available at the NIH sequencing reads archive under BioProject ID PRJNA541842. The source data for figures 4F, 4H, 5E, 5H, 6E, and 6F, Supplementary Figures 6A, 6B, 6G and 7, as well as all western blots are available in the source data file. Code and all other data will be provided upon request.

Field-specific reporting

Please select the one below that is the best fit for your research. If you are not sure, read the appropriate sections before making your selection.

☒ Life sciences ☐ Behavioural & social sciences ☐ Ecological, evolutionary & environmental sciences

For a reference copy of the document with all sections, see nature.com/documents/nr-reporting-summary-flat.pdf

Life sciences study design

All studies must disclose on these points even when the disclosure is negative.

Sample size	Sample sizes were originally determined through a power calculation to have sufficient power to pick up differences in metastastic potential. Due to the failure rate of injection the n of each experiment was varied.
Data exclusions	No data was excluded from this study
Replication	Biological replicates of each study is provided, For quantification replication we had the quantification performed by multiple investigators independently to have a consensus.
Randomization	All mice in the study were age matched and randomly assigned into each cohort
Blinding	Investigators performing the injection were blinded to the identity of the cell type due to the cells being prepped by another researched. For quantifications, all quantifications were made blinded to the cohort of the sample

Reporting for specific materials, systems and methods

We require information from authors about some types of materials, experimental systems and methods used in many studies. Here, indicate whether each material, system or method listed is relevant to your study. If you are not sure if a list item applies to your research, read the appropriate section before selecting a response.

Materials & experimental systems

n/a	Involved in the study	
☐	☒	Antibodies
☐	☒	Eukaryotic cell lines
☒	☐	Palaeontology
☐	☒	Animals and other organisms
☒	☐	Human research participants
☒	☐	Clinical data

Methods

n/a	Involved in the study	
☒	☐	ChIP-seq
☒	☐	Flow cytometry
☒	☐	MRI-based neuroimaging

Antibodies

Antibodies used	The following antibodies were used: COL1A1 [1:1000] (Origene TA309096), CHAD [1:2500] (Abcam ab104757), EGFR [1:1000]

	(CST D38B1), pEGFR [1:1000] (Invitrogen PA5-37553), HSP90 [1:1000] (CST 4874S), Beta-tubulin [1:1000] (CST 2128S), anti-rabbit secondary [1:10000] (Licor 926-32211), anti-mouse secondary [1:10000] (Licor 926-68070).
Validation	Validation for these antibodies are provided on the manufacturer's website

Eukaryotic cell lines

Policy information about cell lines

Cell line source(s)	The PyMT 419 cell lines were a gracious gift from Dr. Stuart Sell and Dr. Ian Guess. The NDL2-5 cells lines were obtained as a gift from Dr. Peter Siegel. The BT-474 cell line was obtained from Dr. Kathy Gallo
Authentication	validated using STR fingerprinting analysis performed at Michigan State University. STR fingerprinting was performed with ProMega Cell ID System run on the ABI 3730xl.
Mycoplasma contamination	Cells were tested for mycoplasm before shipping to the lab and were found to be negative
Commonly misidentified lines (See ICLAC register)	No commonly misidentified cell lines were used

Animals and other organisms

Policy information about studies involving animals; ARRIVE guidelines recommended for reporting animal research

Laboratory animals	The MMTV-Neu and MMTV-PyMT mice were in the FVB background. MMTV-PyMT634 and MMTV-Neu mice were obtained from The Jackson Laboratory
Wild animals	NA
Field-collected samples	NA
Ethics oversight	All animal husbandry and use was conducted according to local, national and institutional guidelines. The study received ethical approval from the Michigan State University Institutional Animal Care & Use Committee (IACUC).

Note that full information on the approval of the study protocol must also be provided in the manuscript.
